# Genomic analysis and antimicrobial resistance profiling of Klebsiella pneumoniae clinical isolates from urinary tract infections in India: prevalence of class A β-lactamase with molecular docking and simulation insights

**DOI:** 10.1099/acmi.0.001200.v3

**Published:** 2026-10-20

**Authors:** Liyana NK, Tusar Mondal, Tobika R, Kumar KM, Yalpi Karthik, Ramakrishna D, Manoj-Kumar Arthikala

**Affiliations:** 1 Department of Bioinformatics, Pondicherry University, Puducherry, India; 2 Ciencias Agrogenómicas, Escuela Nacional de Estudios Superiores Unidad León, Universidad Nacional Autónoma de México (UNAM), León, Mexico; 3 Biotechnology Department, Dayananda Sagar College of Engineering, Dr. C.D Sagar Centre for Life Sciences, Dayananda Sagar Institutions, Bengaluru, India

**Keywords:** antimicrobial resistance, *β*-lactam resistance, *Klebsiella pneumoniae*, molecular docking, molecular dynamic simulation, meropenem

## Abstract

Urinary tract infections are a major global health concern, with *Klebsiella pneumoniae* emerging as a prominent multidrug-resistant uropathogen. The increasing prevalence of extended-spectrum *β-*lactamase–producing strains has reduced the effectiveness of *β*-lactam antibiotics. In this study, 87 clinical *K. pneumoniae* isolates from India were analysed using whole-genome sequencing, MLST and antimicrobial resistance profiling. *β*-Lactam resistance was the dominant phenotype, with blaSHV and blaCTX-M identified as the most prevalent class A *β*-lactamase genes. Molecular docking and 100 ns molecular dynamics simulations were conducted to examine enzyme–antibiotic interactions. Meropenem exhibited the lowest binding affinity with both proteins (−2.695 kcal mol^−1^ for blaSHV and −1.849 kcal mol^−1^ for blaCTX-M), forming unstable complexes, particularly with blaCTX-M. These findings highlight the growing challenge of *β*-lactam resistance and emphasize the need for genomic surveillance and alternative therapeutic strategies against multidrug-resistant *K. pneumoniae*.

## Data Summary

All data supporting the findings of this study are available within the article and its supplementary materials. In accordance with Access Microbiology's Open Data policy, all relevant datasets, including raw and processed data, have been uploaded to the Editorial Manager submission system as supplementary files. These data are accessible to editors, reviewers and readers upon publication. Any additional information required to reproduce the results reported in this study is available from the corresponding author upon reasonable request.

## Introduction

## Introduction

Urinary tract infections (UTIs) are among the most common infectious diseases worldwide and pose serious public health and economic concerns [1]. As one of the leading reasons for antibiotic prescriptions, UTIs are closely associated with the growing prevalence of antimicrobial resistance (AMR), largely due to long-term and uncontrolled antibiotic use [2]. This has led to the emergence of multidrug-resistant pathogens, particularly *Klebsiella pneumoniae (K. pneumoniae*), a common uropathogen of concern due to its propensity to develop resistance. Gram-negative bacteria are the main cause of UTIs, with *Escherichia coli* being the primary pathogen. *K. pneumoniae*, another important pathogen within the Enterobacteriaceae family, also contributes to UTIs alongside various Gram-positive pathogens [1, 3]. The rise in antibiotic resistance among Enterobacteriaceae has resulted in increased mortality rates (40–50%), particularly among critically ill and immunocompromised patients [4].

*K.* pneumoniae is a major reservoir of AMR genes and exhibits a high burden of AMR in India, largely driven by the widespread dissemination of extended-spectrum *β-*lactamase (ESBL)- and carbapenemase-producing strains. Consequently, *K. pneumoniae* has been included in the WHO global priority pathogen list due to its growing clinical importance and the urgent need for new therapeutic strategies [5–7]. Urinary isolates exhibit the highest rates of ESBL production and resistance compared with blood or respiratory isolates [8]. High-risk clones such as ST258 and virulent lineages, including ST23, ST65 and ST147, contribute to outbreaks, severe infections and sepsis through capsular exchange, siderophore production and plasmid-mediated gene transfer [9, 10]. Carbapenem resistance arises via carbapenemases, porin loss and efflux mechanisms, often mediated by mobile genetic elements [11–13].

## Methods

### Data retrieval

Publicly available whole-genome sequencing (WGS) data for *K. pneumoniae* clinical isolates were retrieved from the European Nucleotide Archive (ENA) under BioProject accession PRJEB50614 [14]. This dataset was selected because it comprises clinical UTI isolates collected in India, providing a geographically relevant collection for investigating the genomic epidemiology of AMR in a region with a high burden of multidrug-resistant *K. pneumoniae*. Metadata, including sample accession numbers and sequencing information, were obtained from the ENA database [14]. The project consisted exclusively of paired-end WGS data generated using the Illumina MiniSeq platform. A total of 87 paired-end sequencing datasets were downloaded for genome assembly and subsequent analyses.

### Assembly and annotation

The quality of the raw sequencing reads was initially assessed using FastQC, and summary quality reports were generated with MultiQC [15]. Adapter sequences and low-quality bases were removed using Trimmomatic [16]. High-quality reads were assembled using the CGPS GHRU Assembly Pipeline (https://gitlab.com/cgps/ghru/pipelines/dsl2/pipelines/assembly), a Nextflow-based workflow incorporating SPAdes for *de novo* genome assembly [17]. Assembly quality was evaluated using QUAST and BUSCO [18], and genome annotation was performed using Prokka (v1.14.6) to identify CDSs, tRNA genes, rRNA genes and other genomic features [19].

### MLST analysis

MLST analysis was performed to determine the sequence types (STs) of *K. pneumoniae* isolates. We utilized the MLST pipeline available at https://gitlab.com/cgps/ghru/pipelines/dsl2/pipelines/mlst.git, which is a Nextflow-based implementation of MLST designed to facilitate high-throughput analysis of bacterial genomes. WGS data in FASTA format were processed through this pipeline to identify the seven housekeeping genes typically used in MLST schemes. The pipeline automatically aligned sequences against the *K. pneumoniae* MLST database to assign alleles and determine the ST for each isolate.

### AMR prediction

AMR genes were identified using the ResFinder tool (version 4.1.5) [20], developed by the Center for Genomic Epidemiology [21]. The quality-checked sequences were uploaded to ResFinder in FASTA format. The minimum alignment length and minimum identity thresholds were the default settings for ResFinder. Using the tool, acquired resistance genes that confer resistance to major antibiotic classes, such as *β*-lactams, aminoglycosides, quinolones and carbapenems, were identified. Detected AMR genes were annotated by resistance class and mechanism. Resistance profiles were determined by mapping the detected genes to established AMR mechanisms.

### Molecular docking

Molecular docking studies were conducted to investigate the structural interactions between representative class A *β*-lactamases and clinically relevant *β*-lactam antibiotics. Following AMR profiling, blaSHV and blaCTX-M were selected because they represented the most prevalent class A *β*-lactamase genes identified among the analysed isolates. Meropenem was selected as the focal ligand[22] because it remains one of the most clinically important carbapenem antibiotics for treating severe infections caused by ESBL-producing and multidrug-resistant *Klebsiella pneumoniae*. Evaluating the interaction of meropenem with these prevalent *β*-lactamases provides mechanistic insights into potential resistance-associated structural features that complement genomic resistance profiling. The most prominent class A *β*-lactam resistance proteins identified in AMR studies were modelled using Robetta [23, 24], yielding stable structures validated by the SAVES server [25]. A total of 34 compounds that closely matched the query were selected for docking. The binding sites for target proteins were predicted using the Sitemap tool in Maestro [26].

The grid was formed, the binding site was targeted, and docking scores were obtained using GLIDE (Schrödinger Maestro 2023–4) [27], where the active site grid was formed around key amino acids, and docking scores were obtained. The Protein Preparation Wizard prepared protein structures, and each ligand was minimized using a force field approach. The compounds were converted to 3D using the Maestro workspace and prepared with the Ligand Preparation Wizard, thereby facilitating precise orientation predictions in docking studies.

### Molecular dynamics simulation

Molecular dynamics (MD) simulations were performed on the best protein–ligand complexes identified by docking to assess the dynamic stability of the predicted interactions under simulated physiological conditions. Unlike molecular docking, which provides a static estimate of binding, MD simulations allow assessment of the persistence and stability of protein–ligand interactions over time. These simulations were therefore performed to determine whether the predicted meropenem-*β*-lactamase complexes remained structurally stable and to provide complementary mechanistic evidence supporting the genomic findings. Simulations were conducted using GROMACS (v2023.4) by solving Newton’s equations of motion[28, 29]. Protein and ligand topologies were generated using the CHARMM force field and cgenff[30]. The final complex was simulated under constant temperature and pressure conditions.

## Results

### Genome dataset collection, assembly and preprocessing

A total of 87 paired-end WGS datasets of *K. pneumoniae* clinical UTI isolates were retrieved from the ENA under BioProject PRJEB50614. Metadata, including accession numbers, sample information and sequencing platform details, were obtained from the ENA database. The sequencing datasets were generated using the Illumina MiniSeq platform. Following quality assessment, adapter trimming and genome assembly using the CGPS GHRU Assembly Pipeline, the quality of all genome assemblies was evaluated using QUAST and BUSCO. Based on this re-evaluation, all 87 genome assemblies met the quality criteria and were retained for downstream analyses, including comparative genomic analysis, MLST, AMR profiling, molecular docking and MD simulations. Detailed assembly statistics generated by QUAST and genome completeness assessments obtained using BUSCO for all 87 genome assemblies are provided in Tables S1 and S2, available in the online Supplementary Material, respectively.

### Genomic feature annotation and distribution

Genome annotation of the 87 *K*. *pneumoniae* assemblies was performed using Prokka (v1.14.6). The annotated genomes exhibited a consistent distribution of genomic features across the dataset. On average, each genome contained ~5,212 CDSs, ranging from 4,762 to 5,699 CDSs. The genomes contained an average of 8.8 rRNA genes (range: 6–11), 77.0 tRNA genes (range: 72–86) and 1 tmRNA per genome. The average assembled genome size was ~5.58 Mb, with genome sizes ranging from 5.16 to 5.97 Mb. The number of contigs ranged from 24 to 276, with an average of 87 contigs per genome, reflecting differences in assembly continuity among the analysed genomes. Despite this variation, the overall consistency in genome size, CDS content and RNA gene composition indicates that the genome assemblies were suitable for downstream comparative genomic, AMR and structural analyses. Detailed genome annotation statistics are provided in Table S3.

### MLST analysis

To investigate the population structure and genetic diversity of the clinical isolates, MLST was performed. MLST analysis revealed substantial genetic diversity among the isolates, identifying both known and novel STs based on seven housekeeping genes. ST147 and ST437 were the most prevalent STs, indicating the predominance of these clonal lineages within the study population (Table 1). The identification of diverse and novel STs highlights considerable population diversity and emphasizes the importance of continuous genomic surveillance to monitor the distribution of clinically relevant lineages and their associated AMR determinants.

**Table 1.** MLST analysis of 87 samples based on 7 housekeeping genes

Sample ID	ST	gapA	infB	Mdh	pgi	phoE	rpoB	tonB
ERR10738845	1,710	2	1	2	1	1	10	25
ERR10738778	437	3	3	1	1	1	1	31
ERR10738640	147	3	4	6	1	7	4	38
ERR10738792	Novel*	2	6	1	5	4*	1	639*
ERR10738951	11	3	3	1	1	1	1	4
ERR10738763	147	3	4	6	1	7	4	38
ERR10738685	437	3	3	1	1	1	1	31
ERR10738599	65	2	1	2	1	10	4	13
ERR10738614	20	2	3	1	1	4	4	4
ERR10738890	348	2	1	20	1	12	15	16
ERR10738605	2,096	1	6	1	1	1	46	1
ERR10738784	152	2	3	2	1	1	4	56
ERR10738772	4,843	3	4	6	1	7	273	38
ERR10738776	147	3	4	6	1	7	4	38
ERR10738595	37	2	9	2	1	13	1	16
ERR10738907	14	1	6	1	1	1	1	1
ERR10738798	395	3	1	2	4	1	1	4
ERR10738954	38	2	1	2	1	2	2	2
ERR10738789	Novel*	2	1	2	1	12	1	525*
ERR10738687	147	3	4	6	1	7	4	38
ERR10738804	16	2	1	2	1	4	4	4
ERR10738853	Novel*	2	1	1	1	72	4	33*
ERR10738727	437	3	3	1	1	1	1	31
ERR10738592	231	2	6	1	3	26	1	77
ERR10738591	395	3	1	2	4	1	1	4
ERR10738952	11	3	3	1	1	1	1	4
ERR10738863	11*	3	3	1	1	1	1	4*
ERR10738692	14	1	6	1	1	1	1	1
ERR10738801	147	3	4	6	1	7	4	38
ERR10738904	395	3	1	2	4	1	1	4
ERR10738795	133	12	1	1	2	5	1	36
ERR10738693	15	1	1	1	1	1	1	1
ERR10738717	16	2	1	2	1	4	4	4
ERR10738638	147	3	4	6	1	7	4	38
ERR10738854	101*	2	6	1	5	4*	1	6
ERR10738787	15	1	1	1	1	1	1	1
ERR10738879	Novel*	1	6	1	1	1	46	325*
ERR10738588	2,096	1	6	1	1	1	46	1
ERR10738889	48	2	5	2	2	7	1	10
ERR10738908	29	2	3	2	2	6	4	4
ERR10738793	147	3	4	6	1	7	4	38
ERR10738698	101*	2	6	1	5	4*	1	6
ERR10738689	147	3	4	6	1	7	4	38
ERR10738936	111	2	1	5	1	17	4	42
ERR10738770	Novel	2	1	2	1	2	2	4
ERR10738881	16	2	1	2	1	4	4	4
ERR10738855	Novel*	3	3	1	1	1	1	639*
ERR10738871	3,113	31	1	1	1	3	1	64
ERR10738577	14	1	6	1	1	1	1	1
ERR10738686	437	3	3	1	1	1	1	31
ERR10738758	231	2	6	1	3	26	1	77
ERR10738875	152	2	3	2	1	1	4	56
ERR10738797	2,096	1	6	1	1	1	46	1
ERR10738726	3,113	31	1	1	1	3	1	64
ERR10738694	437	3	3	1	1	1	1	31
ERR10738935	661	4	3	1	36	9	10	14
ERR10738611	661	4	3	1	36	9	10	14
ERR10738610	661	4	3	1	36	9	10	14
ERR10738843	1,710	2	1	2	1	1	10	25
ERR10738938	15	1	1	1	1	1	1	1
ERR10738690	147	3	4	6	1	7	4	38
ERR10738824	2,534	3	1	1	1	3	3	23
ERR10738751	101*	2	6	1	5	4*	1	6*
ERR10738724	105	2	3	2	1	1	4	18
ERR10738697	231	2	6	1	3	26	1	77
ERR10738695	231	2	6	1	3	26	1	77
ERR10738847	1,710	2	1	2	1	1	10	25
ERR10738806	147	3	4	6	1	7	4	38
ERR10738964	54	2	1	1	13	16	15	4
ERR10738691	147	3	4	6	1	7	4	38
ERR10738838	2,370	2	9	1	1	13	1	16
ERR10738590	110*	2	6	1	3	8	1	44*
ERR10738589	231	2	6	1	3	26	1	77
ERR10738937	1,876	2	5	1	1	12	1	18
ERR10738699	38	2	1	2	1	2	2	2
ERR10738771	231	2	6	1	3	26	1	77
ERR10738777	16	2	1	2	1	4	4	4
ERR10738821	45	2	1	1	6	7	1	12
ERR10738888	395	3	1	2	4	1	1	4
ERR10738775	437	3	3	1	1	1	1	31
ERR10738773	111	2	1	5	1	17	4	42
ERR10738696	147	3	4	6	1	7	4	38
ERR10738644	147	3	4	6	1	7	4	38
ERR10738961	16	2	1	2	1	4	4	4
ERR10738712	101	2	6	1	5	4	1	6
ERR10738658	661	4	3	1	36	9	10	14
ERR10738767	101*	2	6	1	5	4*	1	6

* Yellow-highlighted novel (rows 5, 20, 23, 38, 46, 48) indicates new alleles or STs that have not been previously identified in the MLST database. Asterisks (*) denote a novel allele variant at the corresponding locus.

Allelic profiles showed substantial variation among the seven housekeeping genes, with gapA exhibiting alleles 2, 3 and 12 and infB showing alleles 1, 3 and 4, reflecting the genetic diversity present within the sampled *K. pneumoniae* population. The presence of multiple and novel STs indicates the circulation of diverse clonal lineages within the study population. These allelic combinations demonstrate the genetic diversity of the circulating lineages and highlight the value of continuous genomic surveillance for monitoring the dissemination of clinically important clones and AMR determinants relevant to disease management and public health.

### AMR prediction

To characterize the AMR landscape of the study population and identify the predominant resistance determinants, whole-genome sequences were screened using ResFinder. AMR analysis revealed diverse resistance profiles among the isolates, with *β*-lactam resistance genes being the most frequently detected, accounting for 457 total gene-detection events (Table 2). This high frequency reflects the widespread distribution of *β*-lactamase genes among the analysed genomes. Aminoglycoside resistance genes were the second most frequently detected (228 gene detection events), followed by quinolone resistance genes (215 gene detection events). Resistance genes associated with fosfomycin, macrolides, trimethoprim, sulphonamides, rifampicin, tetracycline and phenicol were also identified, indicating the presence of multidrug resistance determinants across the study population (Table S3).

**Table 2.** Distribution of AMR gene detection events identified by ResFinder across *K. pneumoniae* genomes

Resistance type	No. of occurrences
Aminoglycoside resistance	228
Beta-lactam resistance	457
Fluoroquinolone and aminoglycoside resistance	57
Fosfomycin resistance	90
Macrolide resistance	95
Macrolide, lincosamide and streptogramin B resistance	13
Phenicol resistance	31
Quinolone resistance	215
Rifampicin resistance	33
Sulphonamide resistance	71
Tetracycline resistance	21
Trimethoprim resistance	74

Among the *β*-lactamase gene families, blaSHV was the most frequently detected, accounting for 193 total gene detection events, followed by blaOXA (82), blaCTX-M (69) and blaTEM (68) (Table 3). The blaSHV and blaCTX-M gene families comprised multiple allelic variants distributed across the study isolates, reflecting the genetic diversity of *β*-lactamase genes circulating within the population. The predominance of these *β*-lactamase gene families indicates a substantial burden of *β*-lactam resistance determinants among the analysed genomes. Accordingly, subsequent structural analyses focused on the class A *β*-lactamases blaSHV and blaCTX-M to investigate their interactions with meropenem and to generate mechanistic hypotheses regarding *β*-lactam resistance.

**Table 3.** Distribution of *β*-lactamase gene families identified by ResFinder across *K. pneumoniae* genomes

Beta-lactam resistance genes	No. of occurrences
blaCMY	1
blaCTX-M	69
blaDHA	1
blaNDM	1
blaLAP	39
blaOXA	82
blaSFO	3
blaSHV	193
blaTEM	68

Quinolone resistance genes were also highly prevalent and are of particular clinical importance because fluoroquinolones are commonly prescribed to treat UTIs, and resistance to this antibiotic class frequently co-occurs with *β*-lactam resistance in multidrug-resistant *K. pneumoniae*. The study also identified 57 gene detection events associated with both fluoroquinolone and aminoglycoside resistance, together with 13 gene detection events related to macrolide, lincosamide and streptogramin B resistance, highlighting the widespread distribution of multidrug resistance determinants. Collectively, these findings demonstrate the extensive repertoire of AMR genes present among the analysed genomes and underscore the need for continued genomic surveillance, strengthened infection control measures and the development of effective therapeutic strategies to combat AMR.

### Molecular docking analysis

#### 3D protein structure building

To investigate the predicted structural interactions between the predominant class A *β*-lactamases identified through genomic analysis and meropenem, a clinically important carbapenem, molecular docking was performed. The amino acid sequences of blaSHV-106 and blaCTX-M-15 were submitted to the Robetta online server for 3D structure prediction. Among the generated models, the one with the best structural features was selected for further analysis to ensure their accuracy and stability (Fig. 1). These models were subsequently used for docking and interaction studies.

**Fig. 1.**
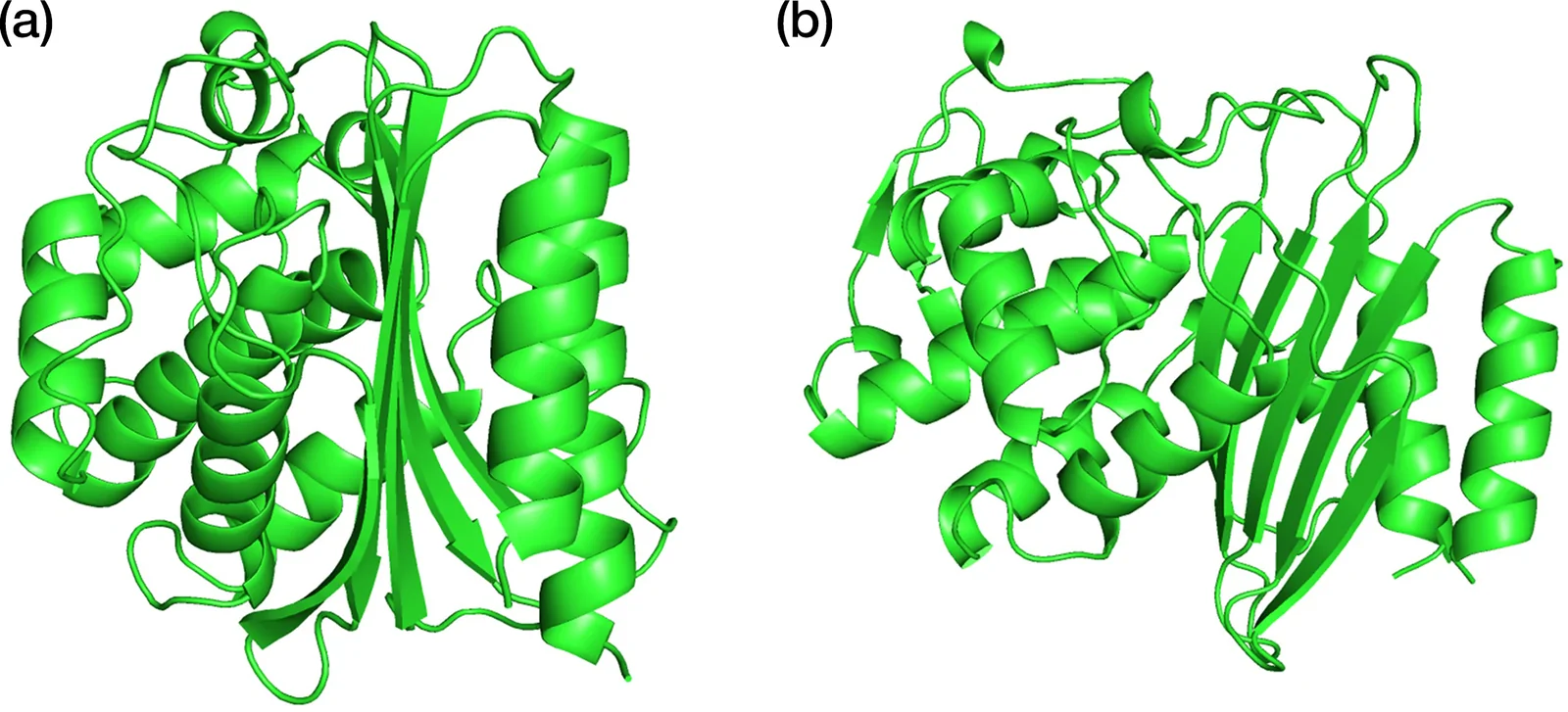
Protein structures: (**a**) structure of blaSHV-106 protein and (**b**) blaCTX-M-15 protein.

#### Binding affinities of meropenem towards class A *β*-lactamases

The binding affinities of meropenem against the class A *β*-lactamases, blaSHV-106 and blaCTX-M-15 (Figs. 2 and 3), were investigated in detail (Table 4).

**Fig. 2.**
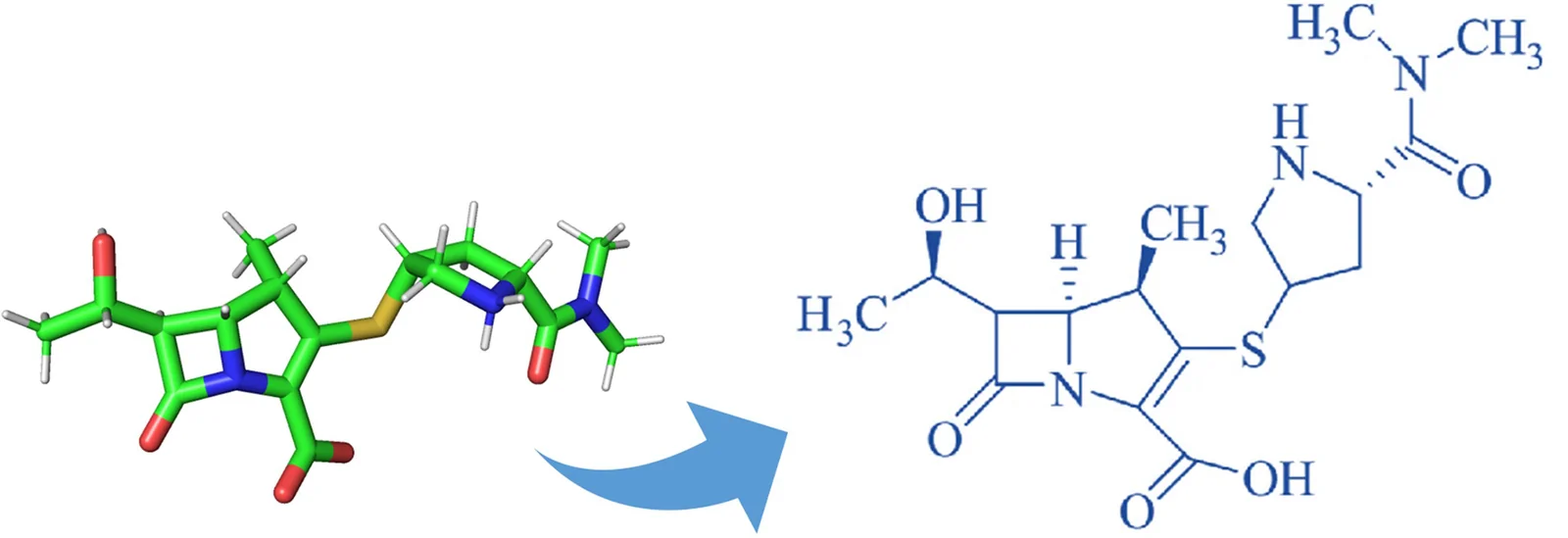
Three-dimensional (left) and two-dimensional (right) structures of the antibiotic meropenem.

**Fig. 3.**
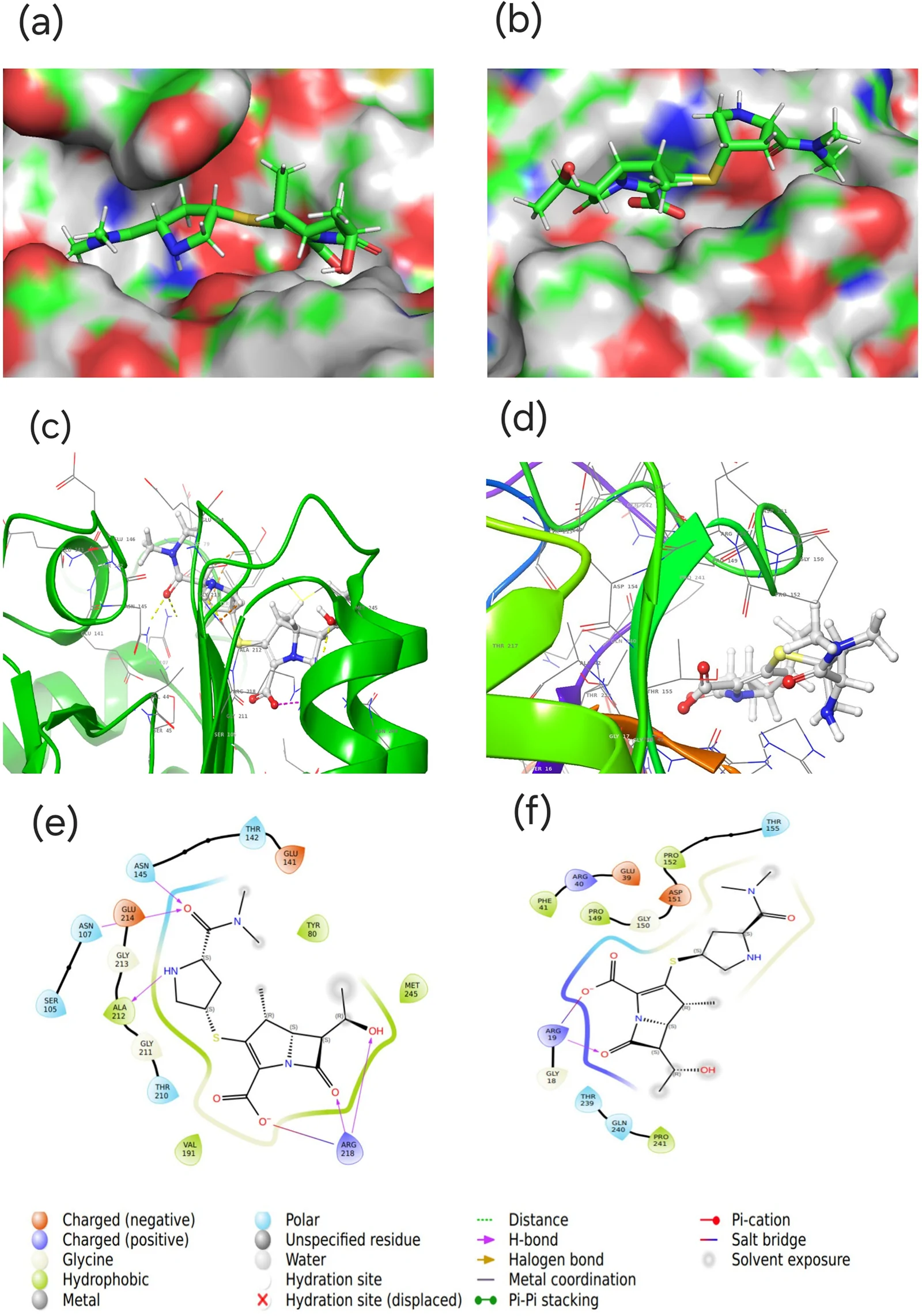
Predicted binding interactions of meropenem with the class A *β*-lactamases blaSHV and blaCTX-M. (**a**) Surface representation of the binding pose of meropenem within the active site of blaSHV. (**b**) Surface representation of the binding pose of meropenem within the active site of blaCTX-M. (**c**) Three-dimensional close-up view of the blaSHV–meropenem complex, highlighting the orientation of meropenem and its interactions with the surrounding active-site residues. (**d**) Three-dimensional close-up view of the blaCTX-M–meropenem complex, illustrating the binding orientation and interactions with neighbouring residues. (**e**) Two-dimensional ligand interaction diagram of the blaSHV–meropenem complex showing hydrogen bonds (HBs) and other non-covalent interactions with active-site residues. (**f**) Two-dimensional ligand interaction diagram of the blaCTX-M–meropenem complex showing HBs and other non-covalent interactions with active-site residues.

**Table 4.** Molecular docking scores of *β*-lactam antibiotics with blaSHV and blaCTX-M *β*-lactamases

Ligands	blaSHV	blaCTX-M
Cefaclor	−6.185	−3.909
Cefadroxil	−5.998	−4.56
Cefamandole	−6.562	−3.69
Cefazolin	−7.118	−4.788
Cefepime	−5.977	−5.042
Cefixime	−5.986	−4.944
Cefmetazole	−5.487	−4.459
Cefoperazone	−6.281	−5.75
Cefotaxime	−5.7	−5.704
Cefotetan	−6.758	−4.548
Cefoxitin	−6.336	−4.661
Cefozopran	−4.979	−6.554
Cefpirome	−5.217	−6.132
Cefpodoxime	−4.997	−4.267
Cefprozil	−5.647	−4.977
Cefquinome	−4.453	−4.621
Cefradine	−5.379	−4.03
Ceftaroline fosamil	−6.01	−4.48
Ceftazidime	−4.644	−6.104
Ceftizoxime	−5.956	−4.699
Ceftobiprole	−6.052	−5.587
Ceftriaxone	−5.583	−3.673
Cefuroxime	−7.243	−5.356
Cephalexin	−6.19	−4.151
Cephalothin	−6.133	−4.807
Cephapirin	−5.677	−5.045
Delafloxacin	−5.313	−5.172
Doripenem	−5.545	−3.883
Enmetazobactam	−3.972	−4.29
Ertapenem	−6.978	−5.086
Imipenem	−5.996	−5.763
Loracarbef	−6.79	−5.29
Meropenem	−2.695	−1.849
Sulopenem	−4.888	−5.243

Post-docking analysis showed low meropenem binding to both blaSHV and blaCTX-M. In blaSHV, Asn145, Asn107, Ala212 and Arg218 formed multiple HBs, with Arg218 dominant, whereas blaCTX-M showed interaction only with Arg19.

The meropenem–blaCTX-M complex exhibited a lower predicted docking score than the meropenem–blaSHV complex and formed fewer HBs (Table 5). While the docking score represents the predicted binding affinity, the number of HBs reflects one aspect of protein–ligand interaction stability. Together, these observations suggest that meropenem adopts a less stable predicted binding pose within the blaCTX-M active site compared with blaSHV.

**Table 5.** Hydrogen bonding residues of blaSHV and blaCTX-M *β*-lactamases with meropenem

Proteins	Total no. of HBs with meropenem	Residues which form HBs with meropenem		
		Residues	No. of HBs	Distance (Å)
blaSHV	6	Asn 107	1	2.55
		Asn 145	1	1.88
		Ala 212	1	2.41
		Arg 218	3	3.15, 2.67, 2.36
blaCTX-M	2	Arg 19	2	3.31, 2.72

#### Comparative docking analysis of class A *β*-lactamases

Molecular docking of 34 *β*-lactam antibiotics against blaSHV and blaCTX-M identified meropenem as having the lowest predicted binding affinity for both enzymes, with docking scores of −2.695 (blaSHV) and −1.849 (blaCTX-M) (Table 4). Because meropenem exhibited the most distinct interaction profile among the screened *β*-lactam antibiotics, the meropenem–enzyme complexes were selected for subsequent MD simulations to evaluate whether the predicted docking poses remained structurally stable under dynamic conditions. Meropenem formed HBs only with Arg19 in the blaCTX-M complex (3.31 Å and 2.72 Å), whereas the blaSHV complex exhibited multiple HBs involving Asn107, Asn145, Ala212 and Arg218, with generally shorter bond distances (Table 5). Together with the lower predicted docking score, the reduced number of hydrogen-bonding interactions observed for the blaCTX-M complex suggests a less stable predicted ligand-binding pose compared with blaSHV. These computational observations provide structural hypotheses regarding protein–ligand interactions and should not be interpreted as direct evidence of AMR or reduced clinical efficacy.

### Molecular dynamic simulation

Molecular docking provides a static representation of protein–ligand interactions. Therefore, MD simulations were performed to evaluate the structural stability of the docked meropenem–enzyme complexes under dynamic conditions. The protein–ligand complexes selected based on the docking analysis were subjected to 100 ns MD simulations to assess the stability of the blaSHV–meropenem and blaCTX-M–meropenem complexes over time. Structural stability was evaluated using root mean square deviation (RMSD), root mean square fluctuation (RMSF), radius of gyration (Rg) and hydrogen-bond analyses.

#### Root mean square deviation

The RMSD analysis (Fig. 4a) highlights the backbone stability of the protein–ligand complexes over a 100 ns simulation. The blaSHV–meropenem complex shows the most stable behaviour, with RMSD values stabilizing at ~0.10–0.15 nm after initial equilibration (0–20 ns), indicating minimal structural deviation. In contrast, the blaCTX-M–meropenem complex exhibits higher RMSD values, reaching ~0.3 nm, with persistent fluctuations around 0.18–0.22 nm. These deviations suggest increased conformational flexibility and reduced stability. Overall, the RMSD results indicate that blaSHV–meropenem maintains greater structural stability than blaCTX-M–meropenem, although both complexes remain within an acceptable nanometre range.

**Fig. 4.**
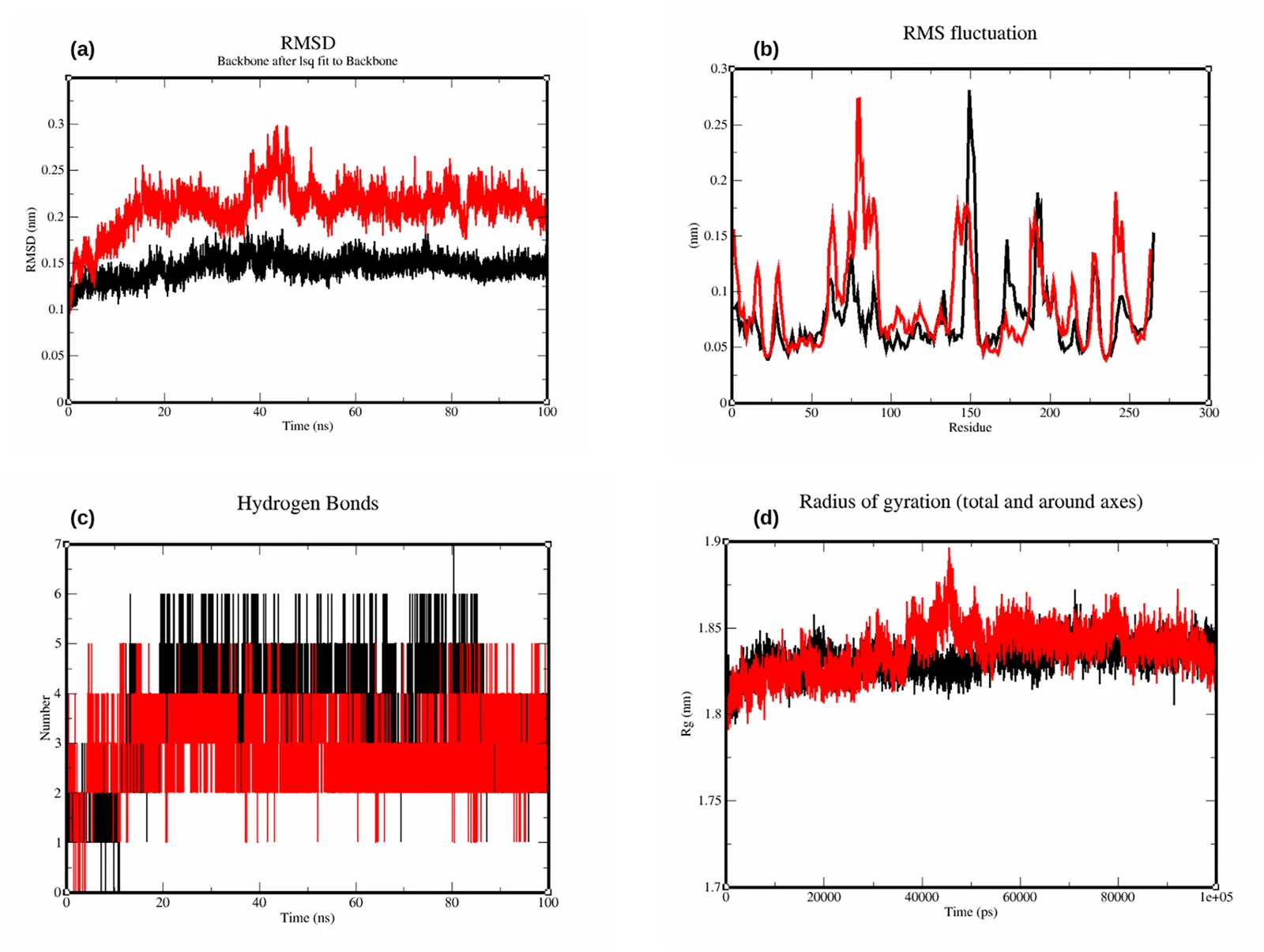
MD simulation analyses of the blaSHV–meropenem (black) and blaCTX-M–meropenem (red) complexes over 100 ns. (**a**) RMSD showing the structural stability of the protein–ligand complexes. (**b**) RMSF showing the residue-wise flexibility of the protein–ligand complexes. (**c**) Hydrogen-bond analysis showing the number of HBs formed during the simulation. (**d**) Rg showing the structural compactness of the protein–ligand complexes throughout the simulation.

#### Root mean square fluctuation

The RMSF plot (Fig. 4b) provides insight into the residue-wise flexibility of the protein–ligand complexes during the molecular dynamic simulation. The blaSHV–meropenem complex (black curve) exhibited relatively low residue fluctuations throughout most of the protein, with RMSF values generally remaining below 0.10 nm, except for a pronounced peak around residues 145–155. In contrast, the blaCTX-M–meropenem complex (red curve) displayed greater flexibility across several regions, particularly around residues 50–100, 135–155, 185–200 and 230–250, where localized increases in RMSF were observed. Despite these regional differences, both complexes exhibited comparatively low fluctuations across most residues, indicating overall structural stability over the 100 ns simulation.

This behaviour indicates a balance between structural stability and flexibility in these regions of the protein. The higher residue-wise fluctuations indicate that this system is more flexible, particularly in these regions, and likely undergoes more conformational adjustments during the simulation. This greater flexibility correlates with the higher RMSD values observed earlier, reflecting a less stable and more dynamic protein–ligand interaction compared to blaSHV.

The RMSF analysis reveals that blaSHV–meropenem maintains a more stable structure than blaCTX-M, while blaCTX-M–meropenem shows localized flexibility. It exhibits significantly higher flexibility, particularly in certain regions, suggesting a less stable and more adaptable structure.

#### HB interaction

HB analysis (Fig. 4c) revealed distinct interaction patterns among the protein–ligand complexes. The blaSHV–meropenem complex consistently maintained 4–5 HBs, indicating a strong and stable interaction that supports structural integrity throughout the simulation. In contrast, the blaCTX-M–meropenem complex showed fewer HBs (2–4) with occasional fluctuations, suggesting a comparatively weaker yet stable interaction. The reduced hydrogen bonding in blaCTX-M correlates with higher RMSD and RMSF values, reflecting greater flexibility and lower complex stability. Overall, the analysis confirms that meropenem forms the most stable and compact complex with blaSHV, whereas binding to blaCTX-M is weaker and more dynamic.

#### Radius of gyration

Rg analysis (Fig. 4d) was used to evaluate the compactness and structural stability of the protein–ligand complexes during the MD simulations. Rg reflects the distribution of atoms around the protein’s centre of mass and indicates conformational stability. The blaSHV–meropenem complex (black curve) exhibited stable Rg values (1.80–1.86 nm) throughout the simulation, indicating early equilibration and sustained structural compactness. In contrast, the blaCTX-M–meropenem complex (red curve) exhibited slightly higher Rg values (1.8–1.9 nm), indicating moderate fluctuations and a less compact structure. Overall, blaSHV–meropenem demonstrated greater structural stability than blaCTX-M.

## Discussion

UTIs are among the most common bacterial infections globally and pose a significant clinical and economic burden, particularly due to escalating AMR. *K. pneumoniae* has emerged as a key uropathogen owing to its remarkable ability to acquire and disseminate resistance determinants, especially against *β*-lactam antibiotics. In this study, WGS, MLST-based population analysis, AMR profiling and *in silico* molecular docking coupled with MD simulations were integrated to investigate resistance mechanisms in Indian clinical isolates. MLST revealed substantial genetic diversity, with ST147 (*n*=13) and ST437 (*n*=6) as dominant STs – both globally recognized high-risk clones associated with ESBL and carbapenem resistance [31]. The presence of novel STs reflects the genetic diversity of circulating clonal lineages within the study population, while the absence of hypervirulent ST23 and ST86 suggests variation in virulence-associated lineages among UTI isolates [32].

AMR profiling revealed that *β*-lactam resistance genes were the most frequently detected determinants of resistance, with the blaSHV and blaCTX-M gene families representing the predominant class A *β*-lactamases, consistent with previous reports from India. Carbapenems remain last-resort antibiotics for the treatment of multidrug-resistant *K. pneumoniae* infections [33], yet the increasing prevalence of carbapenem resistance threatens their clinical efficacy [34, 35]. Molecular docking and MD simulations predicted a weaker, less stable interaction of meropenem with blaCTX-M than with blaSHV, as indicated by higher RMSD and RMSF values and reduced hydrogen-bond stability [36]. These observations are consistent with the increasing prevalence of carbapenem resistance reported in India [37, 38] and provide structural insights into the potential contribution of class A *β*-lactamases to reduced carbapenem susceptibility. Importantly, these structural analyses should not be interpreted as direct predictors of phenotypic AMR or clinical treatment outcomes. Instead, molecular docking and MD simulations provide complementary mechanistic insights into how the predominant *β*-lactamases identified through genomic surveillance interact with meropenem, a clinically important carbapenem, while generating structural hypotheses that require subsequent biochemical and microbiological validation [38, 39]. When integrated with WGS and AMR profiling, these computational analyses strengthen the biological interpretation of resistance determinants and help prioritize candidate enzymes for future biochemical and phenotypic validation. Thus, the structural analyses extend the genomic findings by generating testable hypotheses regarding resistance mechanisms rather than replacing conventional susceptibility testing.

One limitation of this study is that the representative blaSHV and blaCTX-M protein sequences used for molecular docking and MD simulations were not compared with published reference sequences to identify amino acid substitutions. Future studies incorporating comparative sequence analysis and experimental validation may provide additional insights into the structural and functional effects of sequence variation.

## Conclusion

This study integrates WGS, AMR profiling and *in silico* structural analyses to provide a comprehensive assessment of *β*-lactam resistance mechanisms in *K. pneumoniae* clinical isolates from India. The high prevalence of class A *β*-lactamase genes, particularly blaSHV and blaCTX-M, highlights their dominant role in mediating resistance among UTI-associated strains. Molecular docking and MD simulations revealed reduced predicted binding affinity and lower enzyme–ligand interaction stability of meropenem, particularly in complexes with blaCTX-M, consistent with fewer predicted stabilizing non-covalent interactions between meropenem and the modelled *β*-lactamases. These structural analyses complement the genomic findings by providing mechanistic insights into the potential molecular basis of carbapenem resistance rather than serving as direct predictors of clinical resistance. Such mechanistic insights may facilitate the rational design and optimization of future *β*-lactamase inhibitors and support experimental studies aimed at improving therapeutic strategies against multidrug-resistant *K. pneumoniae*. The integration of comparative genomics with structural modelling offers a framework for prioritizing resistance determinants for future experimental validation and surveillance efforts. The identification of novel STs further suggests ongoing evolutionary adaptation under antibiotic pressure. Collectively, this work demonstrates that integrating comparative genomics with molecular docking and MD provides complementary evidence for understanding AMR mechanisms. While these computational findings are hypothesis-generating and require experimental validation, they may assist future genomic surveillance by prioritizing resistance determinants for functional investigation and provide a structural framework for the rational development of next-generation *β*-lactamase inhibitors and improved therapeutic strategies.

## Supplementary material

10.1099/acmi.0.001200.v3 Table S1.

10.1099/acmi.0.001200.v3 Supplementary Material 1.

## Abbreviations

Å: Ångström
AMR: antimicrobial resistance
ENA: European Nucleotide Archive
ESBL: extended-spectrum β-lactamase
FASTA: text-based format for nucleotide or amino acid sequences
GROMACS: GROningen MAchine for Chemical Simulations
HB: hydrogen bonds
MD: molecular dynamics
PRJEB: Project Identifier (European Nucleotide Archive)
Rg: radius of gyration
RMSD: root mean square deviation
RMSF: root mean square fluctuation
STs: sequence types
UTIs: urinary tract infections
WHO: World Health Organization
